# A peptide antigen derived from EGFR T790M is immunogenic in non-small cell lung cancer

**DOI:** 10.3892/ijo.2014.2787

**Published:** 2014-12-01

**Authors:** KAZUYA OFUJI, YOSHITAKA TADA, TOSHIAKI YOSHIKAWA, MANAMI SHIMOMURA, MAYUKO YOSHIMURA, KEIGO SAITO, YASUNARI NAKAMOTO, TETSUYA NAKATSURA

**Affiliations:** 1 Division of Cancer Immunotherapy, Exploratory Oncology Research and Clinical Trial Center, National Cancer Center, Kashiwa, Chiba, Japan; 2 Second Department of Internal Medicine, Faculty of Medical Sciences, University of Fukui, Fukui, Japan; 3 Research Institute for Biomedical Sciences, Tokyo University of Science, Noda, Chiba, Japan

**Keywords:** acquired resistance, CTL epitope, EGFR T790M, immunotherapy, non-small cell lung cancer

## Abstract

Lung cancer is the leading cause of cancer-related deaths worldwide. Epidermal growth factor receptor-tyrosine kinase inhibitors (EGFR-TKIs), such as gefitinib and erlotinib, have demonstrated marked clinical activity against non-small cell lung cancer (NSCLC) harboring activating epidermal growth factor receptor (EGFR) mutations. However, in most cases, patients develop acquired resistance to EGFR-TKI therapy. The threonine to methionine change at codon 790 of EGFR (EGFR T790M) mutation is the most common acquired resistance mutation, and is present in ~50% cases of TKI resistance. New treatment strategies for NSCLC patients harboring the EGFR T790M mutation are required. We evaluated the immunogenicity of an antigen derived from EGFR with the T790M mutation. Using BIMAS we selected several EGFR T790M-derived peptides bound to human leukocyte antigen (HLA)-A^*^02:01. T790M-A peptide (789–797) (IMQLMPFGC)-specific cytotoxic T lymphocytes (CTLs) were induced from peripheral blood mononuclear cells (PBMCs) of HLA-A2^+^ healthy donors. An established T790M-A-specific CTL line showed reactivity against the NCSLC cell line, H1975-A2 (HLA-A2^+^, T790M^+^), but not H1975 (HLA-A2^−^, T790M^+^), and the corresponding wild-type peptide (ITQLMPFGC)-pulsed T2 cells using an interferon-γ (IFN-γ) enzyme-linked immuno spot (ELISPOT) assay. This CTL line also demonstrated peptide-specific cytotoxicity against H1975-A2 cells. This finding suggests that the EGFR T790M mutation-derived antigen could be a new target for cancer immunotherapy.

## Introduction

Lung cancer is the leading cause of cancer-related deaths worldwide (1). Non-small cell lung cancer (NSCLC) accounts for ~80% of all lung cancer cases. Despite recent development in treatment agents, the prognosis for lung cancer patients remains poor (2).

Overexpression of epidermal growth factor receptor (EGFR) is observed in various malignancies, including lung cancer (3). EGFR activation induces many intracellular signaling pathways, such as the mitogen-activated protein kinase (MAPK), phosphatidylinositol 3-kinase (PI3K), and signal transducer and activator of transcription (STAT) pathways, which cause tumor cell proliferation and survival (4). The EGFR pathway is an appropriate target for cancer therapy, and several agents that block this pathway have been developed. In particular, epidermal growth factor receptor-tyrosine kinase inhibitors (EGFR-TKIs), such as gefitinib and erlotinib, demonstrated marked clinical activity against NSCLC harboring an activating EGFR mutation (5–9). However, patients develop acquired resistance to EGFR-TKIs almost without exception (10). A secondary mutation, resulting in a threonine to methionine change at codon 790 of EGFR (EGFR T790M), is the major mechanism of EGFR-TKI resistance (10,11). Additionally, some reports suggest that the EGFR T790M mutation may not be rare and may exist in a small population of in tumor cells before TKI treatment (12–14). Moreover, a pre-existing T790M mutation was associated with shorter progression-free survival (PFS) in patients receiving TKI treatment (13,14). At this time, no standard treatment for EGFR mutant patients with acquired resistance has yet been established, and novel strategies for overcoming this resistance issue are required.

Immunotherapy for NSCLC patients is considered to be a potentially feasible option, because of its high specificity and low toxicity against normal tissues; indeed, several tumor-associated antigen (TAA)-targeted phase 2/3 studies are ongoing (15). However, unfortunately, the results of a TAA-based vaccine therapy study were unsatisfactory (16). One concept for improving the effect of cancer vaccine therapy is to target mutated antigen-derived epitopes. It has been reported that various mutated epitopes were recognized by tumor-reactive T cells (17,18), suggesting that the mutated epitope was potentially immunogenic and thus might function as an immunotherapeutic target. There are few studies of immunotherapy targeting the EGFR T790M mutation. Here, we hypothesized that EGFR T790M-harboring cancer cells could be targeted by activated immune cells, and attempted to assess the immunogenicity of the EGFR T790M mutation-derived antigen *in vitro*. In the present study, we identified the human leukocyte antigen (HLA)-A2-restricted EGFR T790M mutation-derived epitope. Our results suggest that immunotherapy targeting the EGFR T790M mutation-derived antigen may be a novel treatment option for NSCLC patients with the T790M mutation. The combination of immunotherapy and EGFR-TKI therapy also may be a novel strategy for prevention of T790M-mediated resistance.

## Materials and methods

### Cell lines

The human NSCLC cell line H1975 was provided by Professor Seiji Yano (Kanazawa University, Ishikawa, Japan). H1975-A2 (H1975 transfected with HLA-A2) was provided by Dr Tetsuro Sasada (Kurume University, Fukuoka, Japan). Artificial APC-A2 (aAPC-A2) cells, which were generated by transduction of HLA-A^*^02:01, CD80, and CD83 molecules into K562 cells, were provided by Dr Naoto Hirano (Dana-Farber Cancer Institute, Boston, MA, USA). T2 cells (HLA-A^*^02:01, TAP^−^) and human NSCLC cell line 11–18 were purchased from Riken (Saitama, Japan). These cell lines were cultured in RPMI-1640 (Sigma Chemical Co., St. Louis, MO, USA), supplemented with 10% FBS (Gibco-BRL, Carlsbad, CA, USA), 100 U/ml penicillin, and 100 μg/ml streptomycin in a humidified atmosphere containing 5% CO_2_.

### PBMC collection

Peripheral blood samples were collected from four HLA-A^*^02:01-positive healthy donors, after informed consent was obtained. Peripheral blood mononuclear cells (PBMCs) were isolated by density centrifugation using Ficoll-Hypaque (Pharmacia, Uppsala, Sweden) and frozen in liquid nitrogen until use.

### Epitope prediction and synthesis

The epitope prediction software BIMAS (http://www-bimas.cit.nih.gov/molbio/hla_bind/) was used to predict peptides that could bind to HLA-A2. EGFR T790M mutation-derived peptides (purity >95%) were purchased from Scrum, Inc. (Tokyo, Japan). H-2 Kb-restricted ovalbumin (OVA) (257–264) (SIINFEKL) peptide (AnaSpec, Inc., Fremont, CA, USA) was used as a negative control in the peptide-binding assay. HLA-A2-restricted cytomegalovirus (CMV) (495–503) (NLV PMVATV) peptide was used as a positive control peptide, and an HLA-A2-restricted HIV-gag (77–85) (SLYNTYATL) peptide (American Peptide Company, Sunnyvale, CA, USA) as an irrelevant peptide in cytotoxic T lymphocyte (CTL) assays.

### Peptide-binding assay

After incubation in culture medium at 26°C overnight, T2 cells were washed with PBS and suspended in 1 ml Opti-MEM (Invitrogen Life Technologies, Carlsbad, CA, USA) with peptide (100 μg/ml), followed by incubation at 26°C for 3 h and then at 37°C for 2.5 h. After washing with PBS, HLA-A2 expression was measured using a BD FACSCanto II flow cytometer (BD Biosciences, San Jose, CA, USA) using a FITC-conjugated HLA-A2 (MBL Co., Ltd., Aichi, Japan)-specific monoclonal antibody. Mean fluorescence intensity (MFI) was analyzed using the FlowJo software (Tomy Digital Biology Co., Ltd., Tokyo, Japan). An OVA peptide was used as a negative control. A CMV peptide was used as a positive control peptide.

### Generation of DCs

CD14^+^ cells were isolated from PBMCs using human CD14 microbeads (Miltenyi Biotec GmbH, Bergisch Gladbach, Germany). Immature dendritic cells (DCs) were generated from CD14^+^ cells using IL-4 (10 ng/ml; PeproTech, Inc., Rocky Hill, NJ, USA) and granulocyte-macrophage colony-stimulating factor (GM-CSF) (10 ng/ml; PeproTech, Inc.) in RPMI-1640 supplemented with 10% FBS. Maturation of DCs was induced by prostaglandin E2 (PGE2) (1 μg/ml; Sigma Chemical Co.) and tumor necrosis factor-α (TNF-α) (10 ng/ml; PeproTech, Inc.).

### Induction of peptide-specific CTLs

CD8^+^ cells were isolated using human CD8 microbeads (Miltenyi Biotec GmbH) from PBMCs. CD8^+^ cells (2×10^6^ cells/well) were stimulated with peptide-pulsed (10 μg/ml) 100-Gy-irradiated autologous mature DCs (1×10^5^ cells/well) in RPMI-1640 containing 10% heat-inactivated human AB serum. After 1 week, these cells were stimulated twice weekly with peptide-pulsed (10 μg/ml) 200-Gy-irradiated aAPC-A2 cells (1×10^5^ cells/well). Supplementation with 10 IU/ml IL-2 (Proleukin; Novartis, Basel, Switzerland) and 10 ng/ml IL-15 (PeproTech, Inc.) was performed at 3–4-day intervals between stimulations.

### IFN-γ ELISPOT assay

Specific secretion of interferon-γ (IFN-γ) from human CTLs in response to stimulator cells was assayed using the IFN-γ enzyme-linked immuno spot (ELISPOT) kit (BD Biosciences), according to the manufacturer’s instructions. Stimulator cells were pulsed with peptide for 2 h at room temperature and then washed three times. Responder cells were incubated with stimulator cells for 20 h. The resulting spots were counted using an ELIPHOTO counter (Minerva Tech, Tokyo, Japan).

### CD107a assay and generation of a CTL line

CD8^+^ cells isolated using human CD8 microbeads from cultured cells were incubated with peptide-pulsed T2 cells at a ratio of 2:1 for 3.5 h at 37°C. CD107a-specific antibodies (BD Biosciences) were included in the mixture during the incubation period. CD8^+^ CD107a^+^ cells were sorted using a FACSAria II cell sorter (BD Biosciences). Sorted CTLs were stimulated, and the CTL line was established as described previously (19).

### Cytotoxicity assay

Cytotoxic capacity was analyzed using the Terascan VPC system (Minerva Tech). The CTL line was used as the effector cell type. Target cells were labeled in calcein-AM (Dojindo Molecular Technologies, Inc., Kumamoto, Japan) solution for 30 min at 37°C. The labeled cells were then co-cultured with the effector cells for 4–6 h. Fluorescence intensity was measured before and after the culture period, and specific cytotoxic activity was calculated using the following formula: % cytotoxicity = {1 − [(average fluorescence of the sample wells − average fluorescence of the maximal release control wells)/(average fluorescence of the minimal release control wells − average fluorescence of the maximal release control wells)]} × 100%.

## Results

### Assessment of EGFR T790M-derived peptide binding to HLA-A^*^02:01 molecules

As the candidates of HLA-A^*^02:01-restricted EGFR T790M-derived CTL epitopes, we selected five 9- or 10-mer peptides with high predicted HLA-A^*^02:01-binding scores, calculated using BIMAS software. Three of the five EGFR T790M-derived peptides had higher binding scores than the corresponding wild-type peptides. Some studies have reported that modified peptides with single amino acid substitutions exhibit improved affinity for HLA molecules and enhanced immunogenicity (20–22); thus, we also designed two modified peptides. These modified peptides with a substitution of Cys for Val (T790M-D) or Leu (T790M-E) at codon 797 showed higher binding scores (Table I).

**Figure 1:**
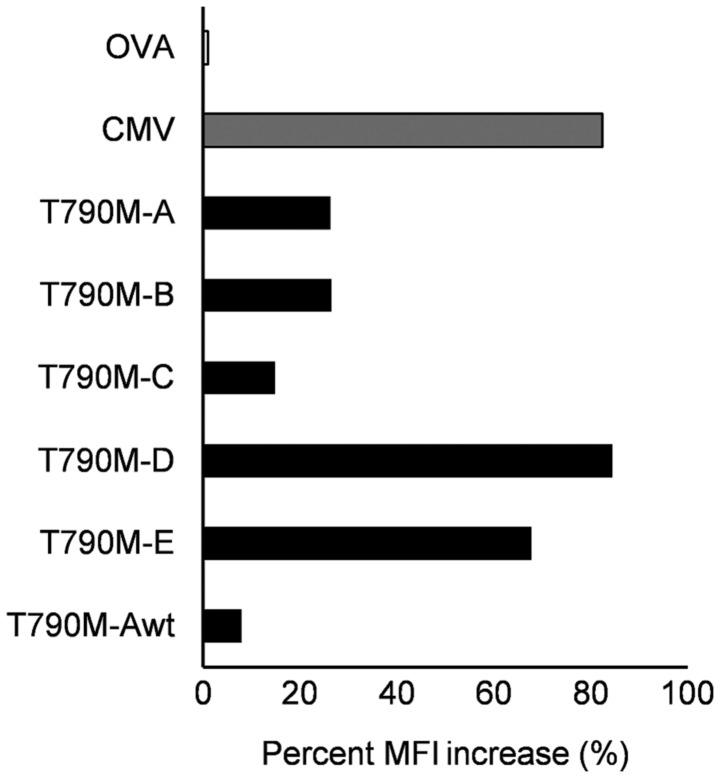
Binding of threonine to methionine change at codon 790 of EGFR (EGFR T790M)-derived peptides to human leukocyte antigen (HLA)-A2 molecule. A T2 binding assay was performed using a FACS system. An ovalbumin (OVA) peptide was used as a negative control. The bars show percent increases in mean fluorescence intensity (MFI). The average of two independent experiments is shown. (Percent MFI increase) = (MFI with the given peptide − MFI without peptide)/(MFI without peptide) × 100.

Using the HLA-A2 TAP-deficient T2 cell line, the binding affinity of the five synthetic peptides to HLA-A2 was assessed. A peptide-binding assay showed that three EGFR T790M-derived peptides were able to bind to HLA-A^*^02:01 molecules. In particular, the binding capability of the T790M-A peptide to HLA-A^*^02:01 molecules was higher than that of the corresponding wild-type peptide. This result suggests that the single amino acid substitution at codon 790 improved the binding affinity for HLA-A^*^02:01 molecules. The binding affinities of two mutated peptides (T790M-D and -E) to HLA-A^*^02:01 were equivalent to that of the CMV peptide used as a positive control (Fig. 1).

### Induction of EGFR T790M-derived peptide-specific CTLs from human PBMCs

**Figure 2:**
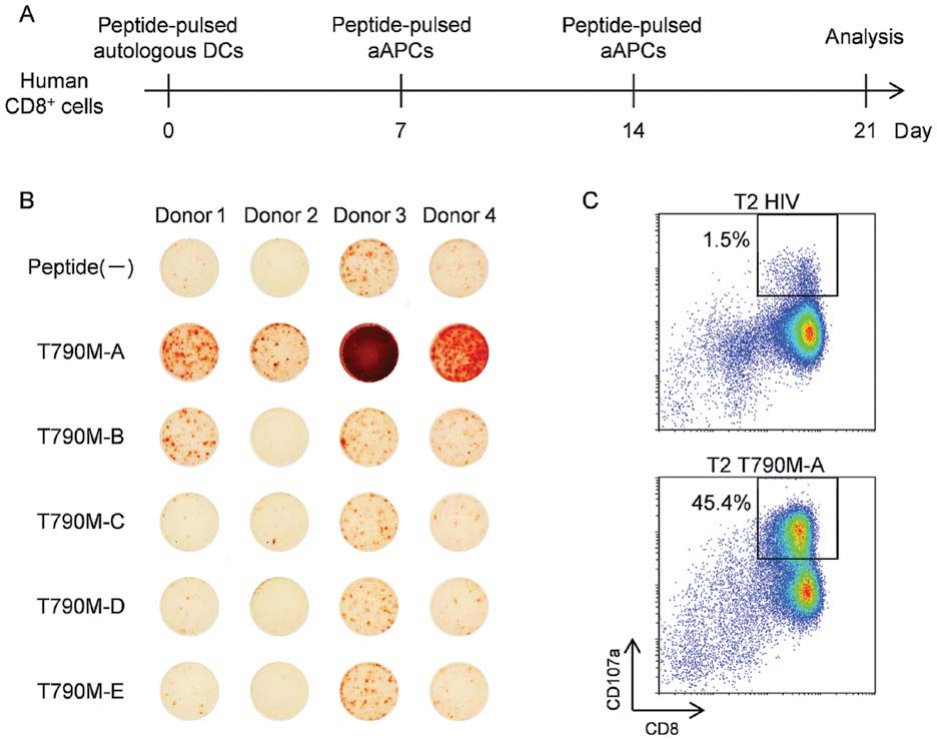
Induction of threonine to methionine change at codon 790 of EGFR (EGFR T790M)-derived peptide-specific cytotoxic T lymphocytes (CTLs) from peripheral blood mononuclear cells (PBMCs) of healthy donors. (A) Induction schedule of peptide-specific CTLs. CD8^+^ cells (2×10^6^ cells) isolated by anti-human CD8 microbeads from PBMCs were incubated with 10 μg/ml peptide-pulsed autologous dendritic cells (DCs) (1×10^5^ cells) on day 0, followed by incubation with 10 μg/ml peptide-pulsed artificial antigen-presenting cell (aAPCs) (1×10^5^ cells) on days 7 and 14. Peptide specificity was assessed by interferon-γ (IFN-γ) enzyme-linked immuno spot (ELISPOT) assay on day 21. (B) IFN-γ ELISPOT assay was carried out (effector, 1×10^5^ cells/well; target, 1×10^5^ cells/well) in duplicate at least three times independently; representative data are shown. (C) T790M-A-specific CTLs of healthy donor 3 were incubated with 10 μg/ml peptide-pulsed T2 cells (E:T = 2:1) for 3.5 h in the presence of an anti-human CD107a antibody. CD8^+^ CD107a^+^ cells were sorted using a FACSAria II cell sorter, which resulted in establishment of a T790M-A-specific CTL line.

To evaluate the immunogenic potential of the five predicted HLA-A^*^02:01-binding peptides derived from EGFR T790M, we attempted to induce peptide-specific CTLs from human PBMCs obtained from four healthy donors. Several reports have shown the usefulness of artificial antigen-presenting cells (aAPCs) for the induction and expansion of peptide-specific CTLs from PBMCs (23,24). Thus, we attempted to induce such CTLs using aAPCs. CD8^+^ cells were isolated from human PBMCs using human CD8 microbeads, and then stimulated with peptide-pulsed DCs for 1 week and subsequently, stimulated twice weekly with peptide-pulsed aAPC-A2 (Fig. 2A). As shown in Fig. 2B, ELISPOT assays revealed that T790M-A (789–797) (IMQLMPFGC)-specific CTLs were induced from PBMCs from all four donors. Also, induction of T790M-B (790–799) (MQLMPFGCLL)-specific CTLs were induced from PBMCs from two of the four healthy donors. However, stimulation with three other peptides, including modified peptides, did not induce peptide-specific CTLs. These results suggest that T790M-A.(789–797) and T790M-B (790–799) have immunogenic potential and that CTLs specific for these peptides can be induced from human PBMCs. Given the effective induction of T790M-A.(789–797) peptide-specific CTLs, we performed further analysis of the T790M-A peptide.

### Generation of EGFR T790M-A-specific CTL line from human PBMCs

Next, we attempted to generate a purified T790M-A (789–797)-specific CTL line. Because the surface mobilization of CD107a is useful for identifying and isolating functional tumor-reactive T cells (25), we performed a CD107a assay to generate a purified T790M-A (789–797)-specific CTL line. Cultured cells stimulated by T790M-A peptide-pulsed DCs and aAPC-A2 *in vitro* were incubated with peptide-pulsed T2 cells at a ratio of 2:1 for 3.5 h at 37°C in the presence of an anti-CD107a antibody. More frequent CD107a^+^ cells were observed when CTLs were co-cultured with T790M-A peptide-pulsed T2 cells compared to HIV-peptide-pulsed T2 cells, and CD8^+^ CD107a^+^ cells were sorted as a purified, peptide-specific CTL line using a FACSAria II cell sorter (Fig. 2C). A purified T790M-A-specific CTL line was established from healthy donor 3.

### Cross-reactivity of the T790M-A-specific CTL line with other EGFR T790M-derived peptides

**Figure 3:**
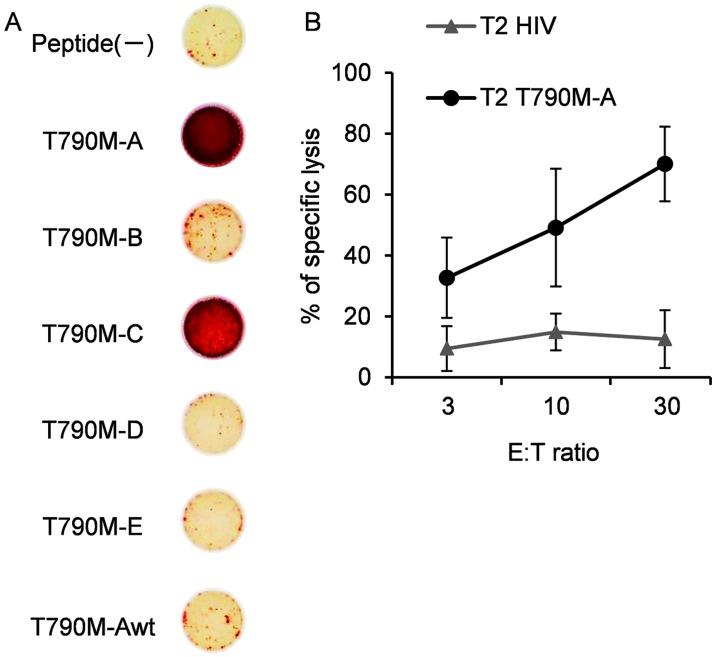
Cross-reactivity of the T790M-A-specific CTL line with threonine to methionine change at codon 790 of EGFR (EGFR T790M)-derived peptides. (A) Interferon-γ (IFN-γ) enzyme-linked immuno spot (ELISPOT) assay against T2 cells pulsed with each peptide. T2 cells pulsed with EGFR T790M-derived peptides (EGFR T790M-A, -B, -C, -D, -E, and Awt) were used as the target (effector 1×10^4^ cells/well, target 1×10^4^ cells/well). The assays were carried out in duplicate wells, and representative data are shown. (B) Cytotoxicity of the T790M-A-specific CTL line against T790M-A peptide-pulsed T2 cells. HIV-peptide-pulsed T2 cells were used as a negative control. Data are presented as means ± SD of three independent batches.

To assess its cross-reactivity with other EGFR T790M-derived peptides, the T790M-A-specific CTL line was cultured with T2 cells pulsed with each peptide, and IFN-γ production was measured by ELISPOT assay. The T790M-A-specific CTL line specifically recognized T2 cells pulsed with T790M-A (789–797) but not non-peptide-pulsed T2 cells. The T790M-A-specific CTL line did not recognize T2 cells pulsed with the T790M-A (789–797) wild-type (ITQLMPFGC) peptide. Also, T2 cells pulsed with T790M-B, -D, and -E were not recognized by the T790M-A-specific CTL line (Fig. 3A). However, the T790M-A-specific CTL line showed cross-reactivity with T2 cells pulsed with T790M-C.

Next, we evaluated the cytolytic activity of the T790M-A-specific CTL line against cognate peptide-pulsed T2 cells. The T790M-A-specific CTL line specifically lysed T790M-A peptide-pulsed T2 cells but not HIV-peptide-pulsed T2 cells (Fig. 3B). These results suggest that the T790M-A-specific CTL line showed cross-reactivity against some EGFR T790M-derived peptides, but not the corresponding wild-type EGFR-derived peptide. This cross-reactivity seems to be favorable for efficacy against EGFR T790M^+^ cancer cells.

### The T790M-A-specific CTL line recognizes and lyses HLA-A2^+^ T790M^+^ NCSLC cells

**Figure 4:**
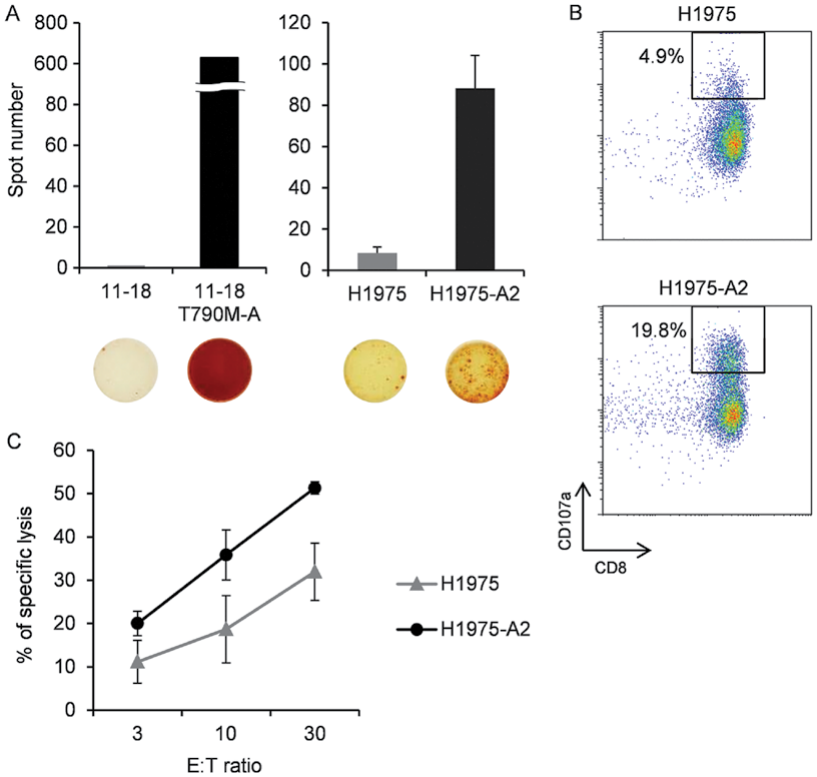
Reactivity of the T790M-A-specific CTL line against non-small-cell lung cancer (NSCLC) cells with or without the T790M mutation. (A) Interferon-γ (IFN-γ) enzyme-linked immuno spot (ELISPOT) assay results for the T790M^+^ and T790M^−^ NSCLC lines. Left: 11–18 and T790M-A peptide-pulsed (10 μg/ml) 11–18 cells were used as the targets (effector 1×10^5^ cells/well, target 1×10^5^ cells/well). Right: H-1975 and H1975-A2 cells were used as the targets (effector, 5×10^4^ cells/well; target, 5×10^4^ cells/well). The bars indicate the IFN-γ ELISPOT counts. (B) CD107a assay of the T790M^+^ NSCLC line (E:T = 2:1). CD8^+^ CD107a^+^ cells were gated. (C) Cytotoxicity against the T790M^+^ NSCLC cell line at the indicated effector/target ratios. Data are presented as means ± SD of three independent batches.

Next, we assessed the ability of the T790M-A-specific CTL line to recognize the HLA-A2^+^ T790M^+^ NCSLC cell line. This CTL line was incubated with 11–18 (T790M^−^, HLA-A2^+^), T790M-A-pulsed 11–18, H-1975 (T790M^+^ HLA-A2^−^), or H-1975-A2 (T790M^+^ HLA-A2^+^), and IFN-γ production was evaluated. We confirmed that the T790M-A-specific CTL line recognized peptide-pulsed 11–18 and H-1975-A2, but not 11–18 and H-1975, cells by IFN-γ ELISPOT assay (Fig. 4A). Similar data were obtained using CTLs from healthy donor 1 stimulated with T790M-A peptide-pulsed DC and aAPC-A2 *in vitro*, which were not purified by the CD107a assay (data not shown).

To evaluate the function of the T790M-A-specific CTL line against H1975-A2, a CD107a assay was performed. CD107a^+^ cells were detected more frequently in culture with H-1975-A2 than with H-1975 cells (Fig. 4B).

Finally, we investigated the cytotoxic activity of the T790M-A-specific CTL line against H-1975-A2. Target cells were labeled with calcein-AM and co-cultured with the effector cells for 4–6 h. The T790M-A-specific CTL line showed cytotoxic activity against H1975-A2 cells, but not H1975 cells (Fig. 4C). These results suggest that the T790M-A-specific CTL line can recognize NSCLC cells harboring the EGFR T790M mutation in an HLA-A2-restricted manner.

## Discussion

Mutated antigens associated with tumor cell progression and survival or drug resistance represent novel targets for cancer vaccine therapy. Warren *et al* evaluated computationally the antigenic potential of somatic mutations that occur in human cancers (26). They showed that several gene mutation-derived epitopes have immunogenic potential, at least computationally. Moreover, point mutations within the ABL kinase domain of the *BCR-ABL* gene are the most common causes of resistance to imatinib in chronic myeloid leukemia (CML) patients (27). Cai *et al* reported that the mutated *BCR-ABL* gene was associated with a TKI-resistance-generated CTL epitope in CML patients (28). These results suggest new immunotherapeutic approaches based on a TKI-resistant mutation-derived neoantigen. That is, mutations associated with acquired resistance to TKI therapy can be targeted by immune-based treatment strategies. This strategy may be an option to treat the gene mutation-mediated drug-resistant cancer cells. In the present study, we demonstrated the immunogenicity of antigens from mutated EGFR that are involved in TKI resistance in NCSLC.

TAAs can be classified into several categories, such as cancer-testis (CT) antigens, overexpressed antigens, differentiation antigens, and mutated antigens. Of these, only mutated antigens are unique, because they are not expressed in normal tissues. Previous reports have shown that peptide vaccine therapy can occasionally induce ineffective CTL responses, contrary to expectations (29–31). One possibility is that the induced antigen-specific CTLs have a low affinity, and thus recognize only target cells pulsed with high concentrations of the peptide and not naturally presented epitopes on tumor cells. Several EGFR-derived CTL epitopes have been identified (32,33); however, the frequency of high-avidity EGFR-specific CTLs seems to be low in patients with EGFR-expressing cancers, because EGFR is a self-antigen that induces tolerance. The ability of low-avidity CTLs to recognize antigen-expressing tumor cells is considered to be weak. However, mutation-derived antigens are not self-antigens; thus, they would not be expected to induce immunotolerance, and so may have high immunogenicity. Indeed, in melanoma patients who experienced dramatic therapeutic effects after adoptive cell therapy with tumor-infiltrating lymphocytes (TILs), the mutated antigen-derived epitope was immunodominant and was recognized by tumor-reactive T cells (34,35).

In the present study, BIMAS was used to select EGFR T790M-derived candidate peptides that bind to HLA-A^*^02:01 according to computer algorithms, and T790M-A-specific CTLs could be induced from PBMCs of all four healthy donors by stimulation with peptide-pulsed DCs and aAPCs. Amino acid substitution of anchor residues (at position 2 and the C-terminus for HLA-A2) can alter the binding affinity (36–38). Leucine and methionine are the preferred anchor residues at position 2 of HLA-A2 (36,37). T790M-A.(IMQLMPFGC) harbors a substitution of threonine to methionine at the anchor site, which confers immunogenicity. Also, valine and leucine are the preferred anchor residues at the C-terminus (36,37).

Then, we designed the modified peptides, T790M-D (IMQLMPFGV, substitution of cysteine to valine at the C-terminus) and T790M-E.(IMQLMPFGL, substitution of cysteine to leucine at the C-terminus). These peptides bound to the HLA-A^*^02:01 molecule strongly (Fig. 1), but could not induce specific CTLs. T790M-D and -E are not self-antigens, being similar in this respect to T790M-A; this difference may be due in part to the difference in the frequency of peptide-specific CTL precursors. To confirm that the predicted candidate peptides are naturally presented peptides on tumor cells, peptide-specific CTL clones or lines induced by the peptides must recognize the tumor cells. A mass spectrometry (MS)-based method facilitates identification of peptide presentation by tumor cells (39). In this study, we confirmed the peptide-specific recognition of tumor cells by a peptide-specific CTL line, but not a CTL clone. However, CTL lines may contain distinct CTL clones that recognize irrelevant peptides, leading to apparent tumor reactivity (40). To avoid misleading tumor recognition and to evaluate the antigen-specific response of a CTL line, we used a peptide-specific CTL line established by CD107a sorting. An IFN-γ ELISPOT assay suggested that the specific CTL line recognized NSCLC cells harboring the EGFR T790M mutation in an HLA-A^*^02:01-restricted manner.

**Figure 5:**
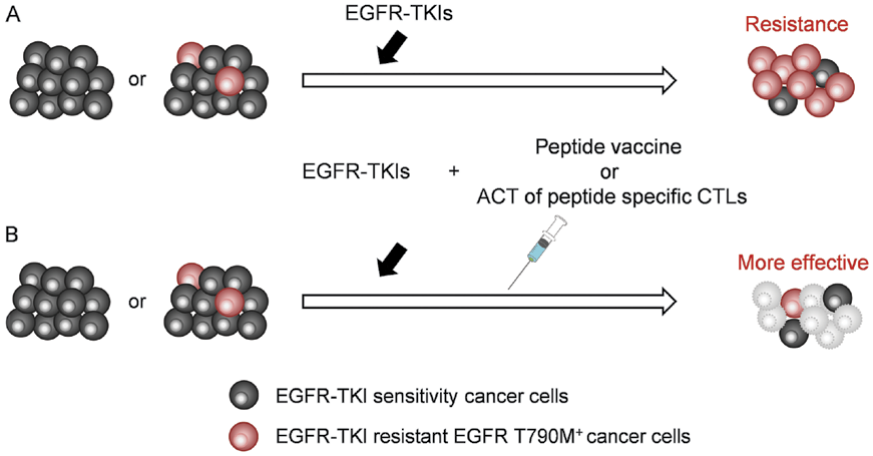
Combination therapy of epidermal growth factor receptor-tyrosine kinase inhibitor (EGFR-TKI) with T790M-targeted immunotherapy. (A) Generally, cancer cells develop acquired resistance to EGFR-TKI. (B) TKI-resistant cells harboring the T790M mutation were targeted by immunotherapy. This combination therapy may be effective against cancers with and without the threonine to methionine change at codon 790 of EGFR (EGFR T790M) mutation.

The T790M-A-specific CTL line did not show activity against the corresponding wild-type peptide. This suggests that EGFR T790M-targeted immunotherapy has no effect on NSCLC prior to EGFR-TKI treatment, with the exception of any pre-existing population of T790M-harboring cells, at least theoretically. Thus, consideration of combination therapy, EGFR-TKI and EGFR T790M-targeted immunotherapy, seems reasonable. Several studies have suggested that combination therapy could improve the efficacy of cancer immunotherapy. For instance, some chemotherapeutic agents can lead to upregulation of TAA expression or improvement of tumor cell resistance to specific CTLs (41). Use of an EGFR-TKI or anti-EGFR antibody augments the IFN-γ-induced expression of MHC classes I and II by A431 malignant human keratinocytes (42). Moreover, gefitinib improved the cytotoxic activity of natural killer cells against H1975 by modulating the interaction between NK cells and cancer cells, and by inhibiting STAT3 expression (43). These results indicate that the combination of EGFR-TKI and immunotherapy may have synergistic activity against NSCLC cells. The concept of combination therapy is shown in Fig. 5. Adding EGFR T790M-targeted immunotherapy to EGFR-TKI treatment could control the progression of cancer cells harboring T790M.

Yamada *et al* reported two HLA-A2-restricted EGFR T790M-derived CTL epitopes (790–799 MQLMPFGCLL and 788–798 LI MQLMPFGCL) (44). In addition to these epitopes, we identified the HLA-A^*^02:01-restricted CTL epitope T790M-A.(789–797 IMQLMPFGC). We found that a T790M-A-specific CTL line established from human PBMCs had the ability to recognize and lyse the HLA-A^*^02:01^+^ T790M^+^ NCSLC cell line, and importantly, did not show cross-reactivity with the corresponding wild-type EGFR peptide. These results suggest that the EGFR T790M-A-specific CTL line recognizes single amino acid substitutions, leading to a low level of auto-immune reaction. The combination of an EGFR-TKI and T790M-targeted immunotherapy may be useful for treatment of NSCLC with the T790M mutation.

## Figures and Tables

**Table I:** Predicted EGFR T790M-derived peptides binding to HLA-A2.

Peptide name	Position	Length	Sequence	BIMAS score^a^
T790M-A	789–797	9	IMQLMPFGC	35.378
T790M-B	790–799	10	MQLMPFGCL	51.77
T790M-C	788–797	10	LIMQLMPFGC	24.921
T790M-D	789–797^b^	9	IMQLMPFGV	495.288
T790M-E	789–797^c^	9	IMQLMPFGL	152.124
T790M-Awt	789–797	9	ITQLMPFGC	0.68

a Binding scores were estimated using the BIMAS software (http://www-bimas.cit.nih.gov/molbio/hla_bind/).

b,c The cysteine (C) residue at position 797 was mutated to valine (V) and leucine (L), respectively. EGFR T790M, threonine to methionine change at codon 790 of EGFR.

## Abbreviations

aAPC: artificial antigen-presenting cell
ELISPOT: enzyme-linked immuno spot
HLA: human leukocyte antigen
IFN-γ: interferon-γ
MAPK: mitogen-activated protein kinase
PBMC: peripheral blood mononuclear cell
PI3K: phosphatidylinositol 3-kinase
PFS: progression-free survival
STAT: signal transducer and activator of transcription
